# Visualization and Treatment of Subclinical Actinic Keratoses with Topical Imiquimod 5% Cream: An Observational Study

**DOI:** 10.1155/2014/135916

**Published:** 2014-05-11

**Authors:** Daisy Kopera, Helmut Kerl

**Affiliations:** Department of Dermatology, Medical University Graz, Auenbruggerplatz 89, 8036 Graz, Austria

## Abstract

*Background*. Imiquimod 5% is licensed for the treatment of external genital warts, superficial basal cell carcinoma, and actinic keratosis (AK) and is being used experimentally in various other dermato-oncological conditions. *Objective*. This observational study shall show that nonmelanoma skin cancer can be detected at its earliest subclinical stage by its reaction with imiquimod and can be cleared by finishing the course of treatment. *Material and Methods*. In this single arm trial 15 patients with chronically sun-exposed skin who had no clinical evidence of AK were treated with 5% imiquimod cream on the face or scalp for 4 weeks three times per week. *Results*. During treatment, all patients developed multiple areas with mild to moderate inflammatory skin reactions, such as erythema, induration, and scaling. Biopsies obtained from 12 patients prior to treatment revealed no malignancies. However, in cases with more pronounced inflammation during treatment, targeted biopsies indicated very early malignant alterations. *Conclusion*. Topical imiquimod treatment of chronically sun-exposed skin without overt clinical signs of AK is able to detect subclinical actinic keratoses (SAK) and to completely clear the lesions, even before they can be clinically diagnosed as AK. In such patients, imiquimod might be able to prevent the evolution of SCC.

## 1. Introduction

Depending on age, lifestyle, and skin type, suqmaous cell carcinoma (SCC) may develop in sun-exposed skin, predominantly on nasal and frontotemporal areas, and on bald male scalps. Actinic keratosis (AK) represents an early or* in situ* SCC [1, 2]. Pathogenesis of AK is explained by potentially carcinogenic UV light interacting with keratinocyte DNA when DNA repair mechanisms fail. AK evolves slowly in the basal layer until they become thicker and clinically evident as coarse erythematous patches in early stages; later they are hyperkeratotic [3–5]. Topical imiquimod 5% cream has been shown to be useful in clearing AK lesions [6–10]. Imiquimod as a toll-like-receptor-7- (TLR-7)-agonist induces cytokines, starting an inflammatory skin reaction directed primarily against malignant or virus-infected cells, but has virtually no effect on normal skin.

Imiquimod 5% cream is licensed in the USA (FDA) and Europe (EMA) for the treatment of external genital warts, superficial basal cell carcinoma, and AK, and is being experimentally used in various other dermato-oncological conditions [11–13]. Imiquimod binds to TLR-7 on monocytes and macrophages indirectly activating intrinsic and acquired immunity due to induction of cytokines with antiviral and antineoplastic abilities. Proinflammatory cytokines subsequently start an inflammatory reaction inducing apoptosis of skin cancer cells. In addition, imiquimod has a direct cytochrome-mediated proapoptotic effect [14, 15].

In photodamaged skin featuring AK, these actions of imiquimod are not restricted to visible AK lesions but often also occur in their vicinity, suggesting that the neoplastic processes are in fact more frequent at a cellular level and not confined to clinically evident lesions, supporting the concept of “field cancerization” [9, 16]. Thus, subclinical actinic keratoses (SAK) exist in an early, macroscopically invisible state and may be exposed visually with imiquimod and treated before they can be diagnosed by usual clinical means and well before potential progression to invasive SCC [17].

Our objective was to investigate the ability of imiquimod to visually expose and, at the same time, also treat clinically invisible SAK. Such early detection and “preventive” treatment might be easier, more effective, and less burdensome than a later treatment of clinically evident cancer.

## 2. Material and Methods

### 2.1. Patients

The local Ethics Committee approved this study. All participants gave informed written consent before the study started and were insured of undesired adverse reactions of the study drug. Healthy male or female volunteers over 60 years of age with no evidence of chronic diseases, presenting with chronically sun-exposed skin on the face or scalp without any clinical evidence for AK lesions, were recruited to the study. Patients with protracted wound healing, abnormal blood clotting or anticoagulation therapy, renal dysfunction, metabolic disturbances, or malignant diseases were excluded.

### 2.2. Treatments and Assessments

Frontotemporal skin was topically treated with imiquimod 5% cream three times a week for four consecutive weeks; this followed the recommended treatment regimen for clinically evident AK [12]. The study drug was applied in the evening and was left on the skin for at least eight hours. Photographic documentation was conducted at baseline, week 2, week 4, and four weeks after the treatment was concluded (week 8). 3 mm punch biopsies were taken from the inconspicious frontotemporal area before treatment in 12 patients. In three patients (nos. 5, 7, and 12), a second biopsy (near the area of the first biopsy) from an erythematous lesion was performed between days seven and fifteen during the treatment phase. A final biopsy of these three patients was taken in the direct vincinity of the previous biopsy at week 8.

## 3. Results

Fifteen patients (3 women, 12 men, age 60 to 86, mean age 70.9 years) were included. Histological specimens before treatment were available from 12 patients.

### 3.1. Clinical Appearance

**Figure 1:**
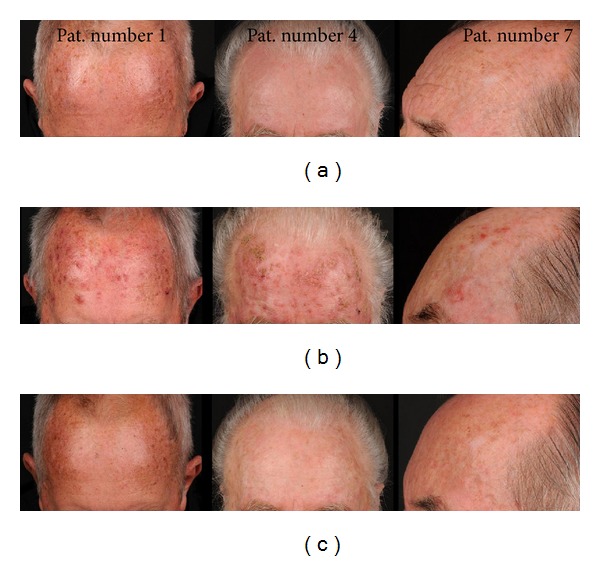
Course of treatment: imiquimod and SAK (patients #1, #4, and #7). (a) Baseline; (b) week 2; (c) follow-up 4 weeks after end of treatment.

Before treatment all patients presented with sun-exposed face, featuring early photoaging (Glogau Type 1) [18] but no AK (Figure 1(a)). During the first two weeks of topical imiquimod treatment all patients developed multiple areas of mild to moderate (none with severe) inflammatory skin reactions clinically featuring erythematous patches and small papules representing very early AK (Figure 1(b)). These areas could therefore be detected and exposed visually although they had been clinically inconspicous at baseline.

After cessation of topical treatment with imiquimod (week 4) the inflammation resolved within three to four weeks leaving no signs of scarring (Figure 1(c)).

As a positive side effect of the treatment in all patients some improvement of the skin quality in the treated area could be observed by the investigators, but this was not evaluated by an objective method such as 3D skin profilometry.

### 3.2. Histopathology

**Figure 2:**
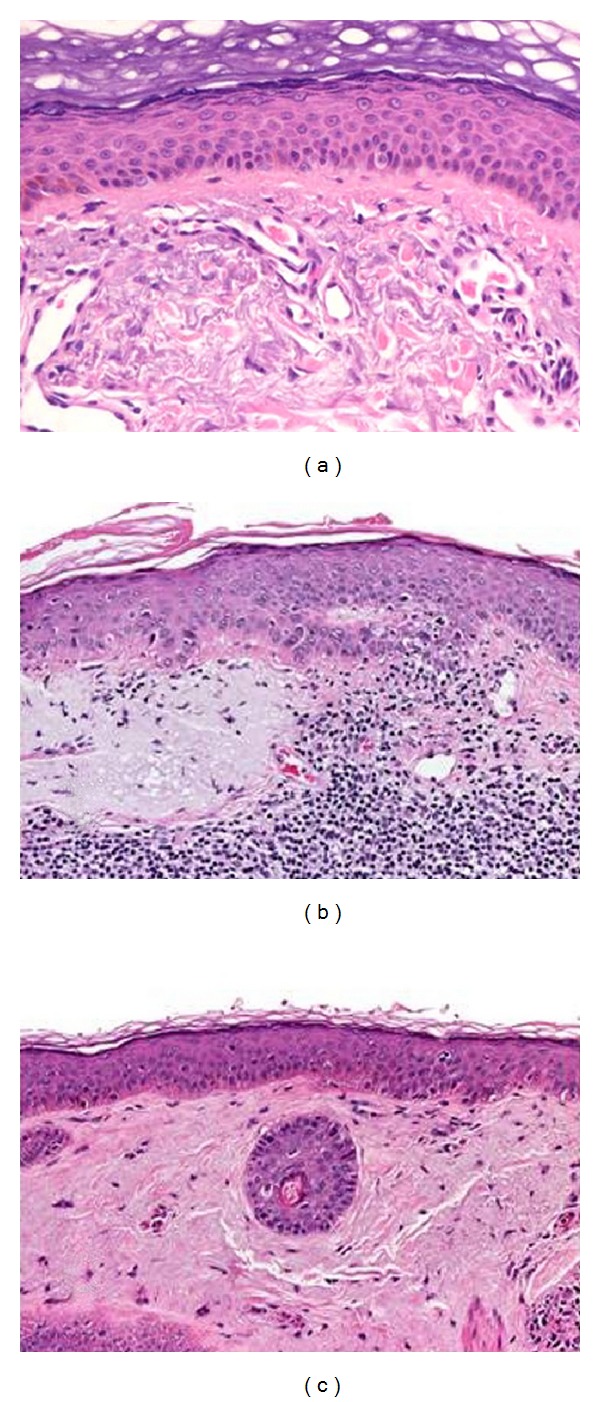
Histopathological examination (patient #7). (a) Before treatment with imiquimod 5% cream (baseline): actinic elastosis is present. No signs of actinic keratosis are recognizable. (b) During treatment with imiquimod 5% cream (day 15): note the early stage of actinic keratosis (AK 1). (c) 4 weeks after treatment with imiquimod 5% cream (week 8): no signs of actinic keratosis can be observed.

Biopsies taken before treatment showed features of sun-damaged skin with marked solar elastosis but no typical signs of AK (Figure 2(a)). The second biopsies from patients 5, 7, and 12 which were performed during imiquimod treatment and taken from inflamed areas revealed slight elongation of rete ridges, focal areas of basal keratinocytes in irregular arrangement, parakeratosis, atypical basal keratinocytes, and dense lymphocytic infiltrates, indicative of actinic keratosis (Figure 2(b)). Biopsies from these three patients taken at the posttreatment visit revealed complete clearance, slight dermal fibrosis, and reorganization of the epidermis (Figure 2(c)).

## 4. Discussion

Photodamaged skin is characterized by mottled pigmentation, thinning, dryness, and wrinkling. Chronic UV exposure leads to cumulative DNA alterations overwhelming physiological DNA repair mechanisms. Consequently carcinogenic transformation in photodamaged skin occurs sooner or later, its extent depending on the skin type and on the amount of accumulated UV exposure.

If chronically UV exposed skin contains very early clinically invisible AKs as precursors of squamous cell carcinoma [8, 17], their existence can be demonstrated visually by triggering an inflammatory reaction in them with imiquimod. For these clinically, yet nonevident, precursors of AK we have coined the term “subclinical actinic keratoses” (SAK).

**Figure 3:**
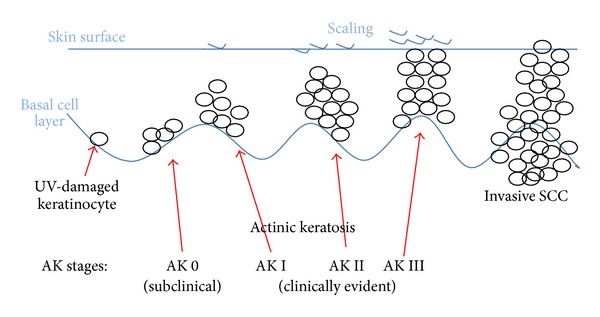
Development of actinic keratosis.

Even when at baseline no diagnostic tool indicated AK; areas of concern promptly showed clinical signs of inflammation during imiquimod therapy. Biopsies taken at this stage featured some signs of premalignant alterations in the histology. The response of atypical keratinocytes to imiquimod in these nonsuspicious areas should not be interpreted as imiquimod-induced alterations but rather as imiquimod-detected altered cells. Clearly AK may exist but may not be evident clinically in their early development as shown in Figure 3.

Similar reactions in clinically normal skin were described as a side effect of systemic 5-fluorouracil (5-FU) therapy as early as 1962 [19]. Several authors have since corroborated these findings [20, 21]. A comparable phenomenon, identified as inflammation of actinic keratoses triggered by systemic 5-FU and other chemotherapeutic agents, was explained as a selective effect of these drugs on atypical keratinocytes in hyperproliferative areas. This was interpreted as a systemic or phototoxic drug eruption [22]. A case featuring similar findings was presented by Sollitto et al. [23], recommending topical steroids for the treatment of these eruptive lesions because they did not consider them representing undiagnosed SAK, but a “recall reaction” at a time when immunomodulation was not yet understood [24]. According to our findings this phenomenon can be better understood as imiquimod not only detects and visually exposes AK but also treats them at the same time.

Topical imiquimod treatment of chronically sun-exposed and eventually photodamaged skin without overt clinical signs for AK is able to detect early malignancies before they can be clinically diagnosed as AK and the subsequent inflammatory reaction usually clears them at this subclinical stage. Giving treatment without an obvious reason may be judged as overdoing, but the issue of overtreatment when applying imiquimod to patients, who do not show skin lesions, can be devaluated by the fact that imiquimod is able to detect subclinical lesions at their earliest stage of development; therefore imiquimod may have a potential role in the prophylaxis of NMSC.
